# Phonological Recoding in Error Detection: A Cross-sectional Study in Beginning Readers of Dutch

**DOI:** 10.1371/journal.pone.0085111

**Published:** 2013-12-30

**Authors:** Eva Van Assche, Wouter Duyck, Robert J. Hartsuiker

**Affiliations:** Department of Experimental Psychology, Ghent University, Ghent, Belgium; Utrecht University, Netherlands

## Abstract

The present cross-sectional study investigated the development of phonological recoding in beginning readers of Dutch, using a proofreading task with pseudohomophones and control misspellings. In Experiment 1, children in grades 1 to 3 rejected fewer pseudohomophones (e.g., *wein*, sounding like *wijn* ‘wine’) as spelling errors than control misspellings (e.g., *wijg*). The size of this pseudohomophone effect was larger in grade 1 than in grade 2 and did not differ between grades 2 and 3. In Experiment 2, we replicated the pseudohomophone effect in beginning readers and we tested how orthographic knowledge may modulate this effect. Children in grades 2 to 4 again detected fewer pseudohomophones than control misspellings and this effect decreased between grades 2 and 3 and between grades 3 and 4. The magnitude of the pseudohomophone effect was modulated by the development of orthographic knowledge: its magnitude decreased much more between grades 2 and 3 for more advanced spellers, than for less advanced spellers. The persistence of the pseudohomophone effect across all grades illustrates the importance of phonological recoding in Dutch readers. At the same time, the decreasing pseudohomophone effect across grades indicates the increasing influence of orthographic knowledge as reading develops.

## Introduction

One of the most important skills that children learn at school is reading. Skilled readers can read without apparent effort and only take a few hundred milliseconds to recognize a word. This highly automated reading process depends on the word representations in a large mental store (the orthographic lexicon). At the same time, skilled readers also know the language’s spelling-to-sound conventions very well. Readers can get from print to meaning by spelling-to-sound translation or by accessing whole-word orthographic representations. These two mechanisms form the basis for the generic dual-route architecture for visual word recognition by models in the Dual-Route Cascaded (e.g., [1] and the Parallel-Distributed Processing tradition (e.g., 2,3). One prominent view is the Dual-Route Cascaded model [1] according to which visual word recognition proceeds via two distinct, but interactive procedures: the lexical and non-lexical routes. In the lexical route, reading relies on the activation of whole-word orthographic and phonological representations. These representations can directly activate semantic representations. Contrary to this lexical retrieval process based on whole-word units, the non-lexical route involves a phonological procedure based on grapheme-phoneme correspondences. It is assumed that these routes interact and that all input words (familiar and unfamiliar (pseudo)words) are processed by both routes in parallel [1]. This reasoning is exemplified in the “two-hoses-filling-a-bucket” concept ([4] according to [5]). 

The connectionist approach of Seidenberg and colleagues (e.g., 2,3) provides another influential view on visual word recognition. It also involves two procedures to get from print to meaning. One procedure goes directly from orthography to semantics, while the other involves a phonological procedure to achieve the same. Harm and Seidenberg [3] presented a model in which meanings are determined by the cooperative division of labor between the direct-visual and phonologically mediated procedures. 

The two mechanisms of phonological and orthographic processing form the basis of several accounts of word reading development (e.g., 6-11). For instance, Ehri [6,7] suggests that English children learn to read words in four phases, starting from pre-alphabetic, over partial and full alphabetic, to consolidated alphabetic when increasingly more sight words are retained in memory. Sight word reading (reading from memory) can only be done for words that we have read before and that are consolidated in lexical memory. In Ehri’s phase theory, alphabetic knowledge that connects graphemes to phonemes is essential to consolidate words in lexical memory. Share [9-11] rather assumes that the act of phonological recoding (connecting graphemes to phonemes) is essential to obtain orthographic knowledge. The self-teaching hypothesis [9,12,13] suggests that phonological recoding, whether overt or covert, is the central process by which beginning readers acquire word-specific knowledge. Each successful recoding provides the opportunity to build up whole-word orthographic knowledge that provides the foundation of skilled word reading. This implies that at a certain point, a child will read the most frequent words via primarily orthographic recognition, whereas less common words are processed phonologically (i.e., item-based phonological recoding [9,14]). 

These theoretical views all posit that phonology plays a prominent role in reading and word comprehension. Also, they assume that children first rely on the recoding of graphemes to phonemes to access semantic word representations before obtaining and using orthographic knowledge of whole-word representations in word comprehension. However, other factors, such as cross-language differences in orthographic consistency and the individual reader’s degree of orthographic knowledge, may determine the extent and speed to which phonological recoding affects word reading in beginning readers. In this respect, it is important to note that the majority of existing studies on phonological recoding in reading were conducted in English, which is a relatively opaque language. Accordingly, there is an overreliance on the English orthography in models of word reading acquisition as noted by Share [15]. It is worthwhile therefore to examine phonological recoding in a variety of languages as benchmarks for future developments in theoretical models of reading. It may be that reading in more transparent languages, such as Dutch, may boost phonological recoding in early reading stages, or speed up the development of other reading strategies, following the logic of Share’s [11] self-teaching hypothesis. Furthermore, it is worthwhile to examine whether children who already acquired orthographic knowledge can also use this knowledge efficiently in a reading task such as proofreading.

## Cross-Language Differences

English studies have shown that phonological recoding strongly influences reading in beginning readers. For instance, in a study by Doctor and Coltheart [16], English-reading children aged six to ten years were asked to judge the meaningfulness of sentences. The youngest group erroneously accepted meaningless sentences that were meaningful when phonologically recoded in most of the cases (e.g., “She *blue* <blew> up the balloon”; “I have *noe* <no> time”), whereas they did not accept meaningless sentences that remained meaningless when phonologically recoded (e.g., “She *know* up the balloon”; “I have *bloo* time”). This pseudohomophone effect became smaller as reading proficiency (age) increased. Doctor and Coltheart concluded that beginning readers rely to a great extent on phonological recoding, and evolve towards using visual representations of words with increasing reading skill.

A strong reliance on phonological recoding in beginning readers has also been found in studies in other languages (e.g., French (e.g., 17,18) and German (e.g., 19,20). For instance, the longitudinal study of Sprenger-Charolles et al. [18] investigated the development of phonological and orthographic processing in French-reading children from grade 1 to the end of grade 4. They used the semantic categorization task in which a higher number of false positive responses on pseudohomophones (e.g., *rouje* for the word *rouge* ‘grey’ in the category color) than on controls is interpreted as a marker of phonological processing (cf. 21,22). From the end of grade 1, pseudohomophone nonwords yielded more false positive responses than controls. The authors hypothesized that the pseudohomophone effect should gradually disappear, but phonological recoding appeared to have a long lasting influence on performance in the semantic categorization task for these French-reading children. A smaller pseudohomophone effect was only observed from the end of grade 3 on.

Similar results were obtained by Grainger, Lété, Bertand, Dufau, and Ziegler [23]. They tested French children in the first to fifth grade and a group of adult readers using a lexical decision task. Grainger et al. tested phonological recoding using pseudohomophones (e.g., *trane*) and orthographic controls (e.g., *trand*), whereas direct whole-word orthographic processing was tested using transposed letter nonwords (e.g., *talbe*) and orthographic controls (e.g., *tarpe*). The results revealed distinct developmental trajectories for the pseudohomophone and transposed-letter effects. Pseudohomophone effects decreased in size, but never disappeared completely, as reading level increased, whereas transposed-letter effects initially increased and then diminished. This implies that beginning readers primarily read via phonological recoding and that this strong reliance on phonological recoding decreases as reading skill and orthographic knowledge develop. 

Many studies have investigated the involvement of phonological recoding in languages such as English or French (e.g., [18,19,23-28], but relatively few studies have investigated phonological recoding in beginning readers of Dutch, although this language is quite often studied in the adult psycholinguistic literature. The English language has a deep orthography with complex grapheme-phoneme correspondences and phoneme-grapheme correspondences, so that a letter may be mapped on different sounds (e.g., the *a* in *have* vs. that in *wave*), while the same sound may be represented orthographically by different graphemes (e.g., the phonetic form [u] in *blue* vs. that in *blew*). But note that there is a high level of consistency at the morphological level [29]. Other languages also have inconsistent grapheme-phoneme correspondences (e.g., Danish), while others are consistent in this respect (e.g., Italian, Dutch, French, German, Spanish). Similarly, in some languages a phoneme can have several spellings (e.g., French, Dutch, Hebrew), while in other languages (e.g., Italian) a phoneme is always spelled in the same way. The language under study here, Dutch, is a fairly regular language, although Dutch phoneme-grapheme correspondences are much less consistent (e.g., the verbs *leiden* ‘lead’ and *lijden* ‘suffer’ have the same pronunciation) than grapheme-phoneme correspondences. Bosman, Vonk, and van Zwam [30] report that pronunciation consistency at the body- rhyme level (i.e., corresponding to what is left of a monosyllabic word after removing the initial consonant or consonant cluster) is 84.5% whereas spelling consistency is 36.8%. When spelling an ambiguous phoneme-grapheme correspondence, the speller needs to know the whole-word orthographic form (e.g., [εi] in *geit* vs. *spijt*) (for a detailed description of Dutch orthography, see 31). Still, Dutch has higher sound-spelling consistencies than French or English. For instance, Bosman et al. [30] reported higher spelling consistency levels at the rhyme-body level in Dutch (36.8%) than in French (2.8%). Because of these differences, it is interesting to examine whether phonological recoding in reading in Dutch, and its evolution as a function of proficiency, may differ from other languages. 

Early studies of Reitsma [32,33] examined the role of orthographic knowledge in Dutch children’s reading. Reitsma [32] showed that beginning readers (7 and 8 years old) can acquire word-specific knowledge quite rapidly; even a few presentations appeared to affect subsequent reading. Although only orthographic learning was examined, Reitsma assumed that beginning readers still rely on phonological recoding to pronounce a word, even though orthographic knowledge also becomes available in word recognition.

A later study of Bosman and de Groot [21] explicitly focused on phonological recoding in the reading of Dutch children in the first grade of elementary school (mean age: 7 years, 4 months). They used a variety of silent reading tasks such as proofreading, lexical decision, and semantic categorization. The critical stimuli were again pseudohomophones (e.g., *wein*, sounding like *wijn* ‘wine’) and control misspellings (e.g., *wijg*). In the proofreading task, children detected fewer pseudohomophones than control misspellings. In the lexical decision task, they erroneously accepted more pseudohomophone misspellings as words than control misspellings. Similarly, in the semantic categorization task, they falsely accepted more pseudohomophone misspellings as category members than control misspellings. Bosman and de Groot’s results show a strong influence of phonological recoding in beginning readers of Dutch (first grade readers), but its development remains an open question. 

A study by Martens and de Jong [34] using the word length effect as another marker effect for phonological processing provided a first investigation of this issue. This word length effect entails that length does not affect reading speed for high frequent words (indicating a lexical reading procedure), whereas longer pseudowords are recognized slower than short pseudowords (indicating a sub-lexical reading procedure).They tested Dutch-reading children in grades 2 and 4 in a lexical decision task. The results indicate that younger children mainly relied on a sub-lexical reading strategy because the second graders were affected by word length when performing lexical decisions, whereas the older fourth graders showed no such word length effect. However, the more extended developmental trajectory of these Dutch beginning readers remains an open question. This will be a key issue for the present paper.

### Orthographic knowledge

Several studies (e.g., 17,21) suggest that individual readers’ orthographic knowledge and reading skill may determine the extent to which phonological recoding affects reading in children. Sprenger-Charolles et al. [17] studied French-reading children from kindergarten until the end of grade 2 to examine phonological recoding in a silent reading task and to examine the role of phonological recoding in the construction of the orthographic lexicon. The use of phonological recoding was assessed in a semantic categorization task with pseudohomophone (e.g., *pome* derived from *pomme* for the food category) and visual foils (e.g., *pomne*). Based on the results of an orthographic choice task at the end of grade 2, pupils were categorized into either an expert or a poor spellers group. There were no differences in the processing of homophone and visual foils at the middle of grade 1 between both groups. However, at the end of grade 1, only the future expert spellers showed a pseudohomophone effect (i.e., they correctly classified pseudohomophone misspellings as non-exemplars less often than control misspellings) in the semantic categorization task, while both groups showed effects at the end of grade 2. Sprenger-Charolles et al. suggested that the use of phonological mediation in early reading acquisition is a mechanism allowing readers to construct an orthographic lexicon (cf. 9). However, the results of the poor speller group should be handled with caution as these results were based on only 7 subjects (compared to 19 children in the expert speller group).

The Dutch study of Bosman and de Groot [21] discussed earlier also compared the performance of more and less advanced readers. They used the results of this comparison, and the results of the influence of base-word frequency, to test the verification hypothesis (e.g., 22,35,36). This hypothesis assumes that a pseudohomophone can activate the spelling of the base word and that orthographic knowledge can be used to verify the spelling of the presented pseudohomophone. It provides an alternative interpretation for the effect of reading skill as given in other studies (e.g., 16). For instance, Doctor and Coltheart [16] suggest that better readers differentially use phonological recoding in word processing with a more frequent use of a direct lexical access procedure. Bosman and de Groot showed that in the majority of the experimental tasks, the more advanced readers detected more pseudohomophones as incorrect words than the less advanced readers did, but both groups scored equally on the control misspellings. They attributed these differences between more and less advanced readers to a more efficient spelling verification process in more advanced readers. Interestingly, the results also showed that spelling verification is not yet stable in beginning readers, as the performance of the more advanced readers dropped to that of the less advanced ones when the critical stimuli in the proofreading task were presented in stories as opposed to lists of unconnected words. Similarly, in the semantic categorization task, there was no effect of reading level. These results show that the task context can provide constraints that work against successful verification. With regard to the role of base-word frequency, it was assumed that the spellings of high-frequency words are more available to verification than those of low-frequency words, but the base-word frequency manipulation did not yield any effects. Nevertheless, these results show that individual reading skill influences reading acquisition. 

### The present study

Based on this literature overview, the present study has the following aims. In Experiment 1, we aimed at investigating the development of phonological recoding in beginning readers of Dutch. We used the pseudohomophone effect in the proofreading task (cf. 21) to investigate phonological recoding in silent word reading in different age groups (children in primary school, grades 1 to 3, aged 7 to 9). Given that the reading acquisition accounts of Share [9,10] and Ehri [6,7] predict that phonology plays an essential role in beginning reading, we predict that Dutch children will detect fewer pseudohomophone misspellings than controls. With increasing reading experience and increasing orthographic knowledge, we expect that the performance on pseudohomophone misspellings will improve, but not equal performance on control misspellings following similar findings in other languages such as English [24,26] or French [18]. Furthermore, reading in the Dutch language, which has a higher sound-spelling consistency than French or English (e.g., 30,37), may boost phonological recoding in early reading stages so that, following the logic of Share’s [11] self-teaching hypothesis, a strong reliance on phonological recoding may lead to the fast development of orthographic knowledge. 

Experiment 2 aimed at investigating how individual’s degree of orthographic knowledge modulates phonological recoding in reading. Using the same proofreading task as in Experiment 1, we tested different age groups of more and less advanced spellers (children in primary school, grades 2 to 4, aged 8 to 10). We predicted weaker effects of phonological recoding for more advanced spellers, as reflected by a smaller pseudohomophone effect. 

In addition to the proofreading task in Experiments 1 and 2, an orthographic choice task (Experiment 1) (cf. 18,21) or spelling test (Experiment 2) was used to test whether children actually knew the spelling of the words from which the pseudohomophones were derived. An important methodologically new aspect of our study was that the results of the orthographic choice task were used to discard items from the proofreading data for which participants simply did not know the correct spelling. So, any effect of phonological recoding in the proofreading task indicates the strong impact of phonological recoding because children actually knew the words’ spellings in the orthographic choice task or spelling test. This procedure of removing unknown words from analyses was not adopted in the previous studies by Bosman and de Groot [21] and Sprenger-Charolles et al. [17,18] and this could have biased their results because readers may by definition only rely on phonological recoding if the correct spelling of a word is not known. Importantly, Starr and Fleming [28] have shown that removing commonly misspelled homophones from the analyses resulted in the reduction of homophone confusion error rates by approximately half in their Experiment 4. If children did not know the correct spelling of a given target, the false acceptance of the derived pseudohomophone as a real word in a semantic categorization task could have originated from the activation of a (wrong) orthographic lexical code, and not necessarily from phonological recoding. 

## Experiment 1

### Method

#### Participants

Fifty-four children of a primary school in Eastern Flanders, Belgium, participated: 20 children (of which 10 female) of grade 1 (mean age: 6 years, 10 months), 17 (10 females) of grade 2 (mean age: 8 years) and 17 (9 females) of grade 3 (mean age: 8 years, 8 months). Written informed consent was received from all children’s parents and the study was approved by the local ethical committee at the Ghent Faculty of Psychology and Educational Sciences. The children were all native speakers of Dutch who received formal reading instruction from around six years when they attended the first grade. In Belgium, children start the first grade in September based on their date of birth year. At the time of testing, they had received about 25 weeks of formal reading and writing instruction. 

All children received the same reading curriculum *Taalsignaal* (Wolters Plantyn). This specific reading and language method is mandated by the school, but not by the government. In Belgium, schools can choose freely which textbooks they use on the condition that the textbooks comply with the final attainment levels for each grade mandated by the government. The *Taalsignaal* books are used in every grade and offer a general framework which is used for learning to read, write, listen and speak. This means that children of grades 2 and 3 had been instructed according to the same method (and teacher) when they were in grade 1. There were no significant differences between grades on the mean writing and spelling report scores of the month before testing as shown by an independent samples t-test (*p* > .16). This indicates the comparability of the three age groups, excluding base proficiency confounds effects when comparing grades. 

Reading didactics in grade 1 start by learning to read some simple words as a small vocabulary (e.g., the names *Leen*, *Rik*, *Mop*, *Jan*, and the words *mus* ‘sparrow’, *ik* ‘I’, *zie* ‘see’, *en* ‘and’, *klas* ‘class’, *muur* ‘wall’, *raam* ‘window’). At the same time, children learn the sound of each letter individually. So, early reading didactics both relies on whole-word forms and on converting letters to phonemes. 

#### Stimuli

We selected twelve monosyllabic base words with a mean length of 4.3 letters (*SD* = 0.65) from the first three reading books of the first grade. Children of the first grade were instructed in reading and spelling the words in these books and were all proficient in reading the books. For each base word, a pseudohomophone and a control misspelling were created. The pseudohomophones shared the phonology of the base word, in contrast with the visual control mispellings. We adopted eight base words and their corresponding pseudohomophones and control misspellings from the stimuli of Bosman and de Groot [21]. This increases comparability across (Dutch) studies. In all of the selected base words, a particular phoneme can be mapped to several graphemes. Examples of these mappings are presented in Table 1. 

**Table 1:** Examples of the phoneme-to-grapheme mappings of the selected base words.

**Sound**	**Letters**	**Examples**
[x]	g, ch	weg (road), nacht (night)
[εi]	ij, ei	wijn (wine), kijkt (he looks), klein (small), geit (goat)
[t]	t, d	hoed (hat), koud (cold),
[Au]	ou, auw	blauw (blue), kous (sock), zout (salt)
[f]	v, f	geeft (he gives)

To ensure that any effect of our phonological manipulation was not confounded with visual similarity between the nonword and the base word, it was essential to control this parameter across conditions. Therefore, we used the Orthographic Similarity (OS) index of Van Orden [22] to check orthographic similarity between pseudohomophones and base words on the one hand, and between control misspellings and base words on the other. Van Orden [22: p. 196] defines graphemic similarity (GS) between two letter strings as GS = 10([(50F + 30V + 10C)/A] + 5T + 27B + 18E) with *F = number of pairs of adjacent letters in the same order, shared by pairs, V = number of pairs of adjacent letters in reverse order, shared by pairs, C = number of single letters shared b word pairs, A = average number of letters in two word pairs, T = ratio of shorter word to longer word, B = if first two letters are the same B* = 1 *else B* = 0*, E = if last two letters are the same E = 1 else E* = 0. Then, Van Orden [22] calculates Orthographic Similarity by determining the ratio between the GS of word 1 with itself and the GS of word 1 and word 2.This index ranges on a scale from 0 to 1, where 0 indicates no orthographic similarity between the items and 1 full orthographic overlap. Mean OS between pseudohomophones and base words (*M* = 0.68, *SD* = 0.07) did not differ significantly from mean OS between control misspellings and base words (*M* = 0.70, *SD* = 0.06) as shown by a paired samples t-test (*p* > 0.28). Thus, pseudohomophones and controls were equally ‘wordlike’, relative to the base word. Furthermore, the pseudohomophones and control misspellings did not differ from each other with respect to word length (number of letters), neighbourhood size [38] and bigram frequency (another measure of word likeness of letter strings in a given language, see 39) (all paired samples t-test ts < 1). These variables were computed using the WordGen stimulus generation software [39], on the basis of the CELEX lexical database [40]. This matching procedure ensured that the critical difference between pseudohomophones and controls is the fact that pseudohomophones, although not written as real words, do sound like real words. The base words, pseudohomophone and control misspellings are listed in the Appendix S1. 

Two lists of 60 items each were created. Each list contained the same 48 correctly spelled monosyllabic filler words (similar word length, *M* = 4.2 letters, *SD* = 0.72), also selected from the first three reading books of the first grade. Additionally, each list contained 6 pseudohomophone and 6 control misspellings of the same pair (so that each child saw both the pseudohomophone and control misspelling of a pair). Stimulus lists were presented in two blocks of 30 trials to avoid concentration issues for the children in grade 1. 

#### Procedure

The written test was administered simultaneously to all the children of a class. Children could not see each other’s forms. Instructions emphasized to read the page very carefully and to mark each misspelling (both nonwords and wrongly spelled words) they came across. We asked the children to pretend to be a teacher correcting lists of words. Instructions were repeated several times and the children had the opportunity to ask for clarification before the experiment started. 

Each child received the first and second block of one of the two stimulus lists. The first and second block of each main list contained the same correctly spelled filler words. Children who sat next to each other received different lists. Between blocks, there was a short break of about half an hour. After the completion of the second block of the proofreading task, all children completed an orthographic choice task, in which each pseudohomophone and base word pair was presented on a sheet of paper. The children were instructed to mark the incorrectly spelled item from each pair. 

### Results

#### Orthographic choice task results

Accuracy was analyzed in analyses of variance (ANOVAs) with Grade (three levels: grade 1, 2, and 3) as the independent variable. Analyses were carried out with participants (F_1_) and items (F_2_) as the random variables. The results showed that most children knew the spelling of the base words. Mean accuracy was 0.78 (SD = 0.19) in the first grade, 0.93 (SD = 0.09) in the second grade and 0.95 (SD = 0.11) in the third grade. Children performed significantly above chance in each grade (all *p*s < .001). In grade 1 however, there were 4 children who were performing at chance level. There was one item for which scores were lower than chance level in grade 1 (base word *koud*). Accuracy scores improved between grades [*F*_1_(2,51) = 8.61, *p* < .001; *F*_2_(2,22) = 6.85, *p* < .01]. Planned comparisons showed that the improvement was significant between the first and second grade [*F*_1_(1,51) = 10.60, *p* < .01; *F*_2_(1,11) = 6.06, *p* < .05] but not between the second and third grade [*F*s < 1]. 

#### Proofreading results

**Figure 1:**
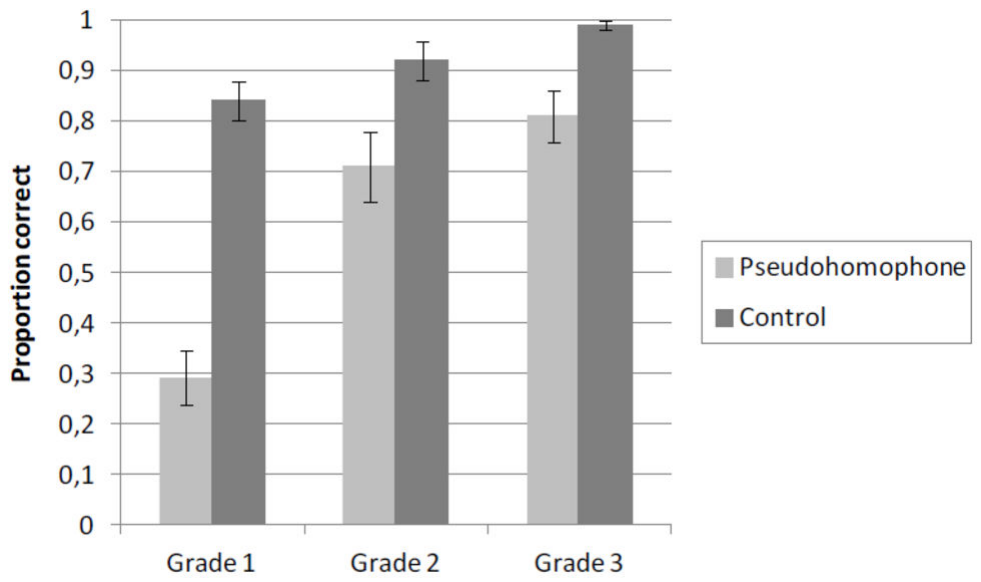
Mean proportion correctly classified pseudohomophone and control misspellings as a function of Grade in Experiment 1. Error bars show standard errors. Proportions are based on 6 items per condition.

An ANOVA was run with Grade (three levels: grade 1, 2, and 3) and Word type (two levels: pseudohomophone vs. control) as the independent variables, and accuracy as the dependent variable. For each child and stimulus, we verified whether the correct spelling of that specific word was known by the child (as determined in the orthographic choice task). If this was not the case, the pseudohomophone and its corresponding control misspelling were removed from the data (cf. 28). Following this procedure, 25% of trials were removed in grade 1, 11% in grade 2, and 4% in grade 3. Analyses were carried out with participants (F_1_) and items (F_2_) as the random variables. Participant and item means of the proportion correctly identified misspellings were calculated. Mean accuracy by Word type and Grade is presented in Figure 1. 

Performance of the children improved between grades, as indicated by a significant main effect of Grade [*F*_1_(2,51) = 25.54, *p* < .001; *F*_2_(2,44) = 32.82, *p* < .001]. The main effect of Word type was also significant [*F*_1_(1,51) = 59.87, *p* < .001; *F*_2_(1,22) = 58.91, *p* < .001]. Control misspellings were detected more often than pseudohomophone misspellings. This pseudohomophone effect was significant in each grade: grade 1 [*F*_1_(1,51) = 69.87, *p* < .001; *F*_2_(1,22) = 81.09, *p* < .001]; grade 2 [*F*_1_(1,51) = 8.37, *p* < .01; *F*_2_(1,22) = 8.41, *p* < .01]; grade 3 [*F*_1_(1,51) = 6.07, *p* < .05; *F*_2_(1,22) = 9.96, *p* < .01]. Importantly, there was a significant interaction between Grade and Word type [*F*_1_(2,51) = 9.41, *p* < .001; *F*_2_(2,44) = 14.22, *p* < .001]. Differences in detecting pseudohomophone and control misspellings were more pronounced in the first grade (0.29 vs. 0.84) than in the second or third grade (0.71 vs. 0.92 and 0.81 vs. 0.99, respectively). Planned comparisons showed that the pseudohomophone effect was significantly stronger in the first than in the second grade [*F*_1_(1,51) = 12.53, *p* < .001; *F*_2_(1,22) = 18.02, *p* < .001]. The planned comparison between the second and the third grade was not significant [*F*s < 1]. 

Taken together, the results from Experiment 1 show that in each grade, children detected more control misspellings than pseudohomophone misspellings. This pseudohomophone effect was more pronounced for children in the first than in the second grade, but it did not decrease through grade 3. An orthographic choice task, on the basis of which spelling errors were filtered out from the main task, ensured that these effects were not confounded with insufficient spelling knowledge of the base words. 

## Experiment 2

To examine the role of increasing orthographic knowledge on the pseudohomophone effect, Experiment 2 was conducted in which more and less advanced spellers in grades 2, 3, and 4 were tested. In each grade, we split the group into children with below average and above average orthographic knowledge based on their spelling scores. As readers become more proficient, they have more word-specific knowledge and they grow less dependent on phonological processes. The prediction follows that more advanced spellers should detect more pseudohomophone misspellings than less advanced spellers, as they have better orthographic knowledge.

Next to this main theoretical objective, conducting an additional experiment allowed us to replicate the decrease in pseudohomophone effects with age while improving the methodology in several ways. First, even though filtering out unknown words in the proofreading analyses, based on the orthographic choice task, already provided a methodological improvement compared to previous studies that did not remove unknown words, children who were unsure about the spelling, would still have the correct item 50% of the time by guessing. We therefore used a straightforward spelling test as a measure of orthographic knowledge in Experiment 2 (cf. 16,28). Second, the orthographic choice task was administered right after the second part of the proofreading task and although this was also the case in other studies (e.g., 17,18,21), this might have influenced response rates because subjects could be primed by the presence of the stimuli in the proofreading lists. Therefore, in Experiment 2, the spelling test was administered several hours after the proofreading task. Also, children had to proofread lists that contained all the pseudohomophones and control words, while participants only saw half of the stimuli in Experiment 1. Finally, larger subject groups were used in order to split up the children of each grade in a group of more and less advanced spellers. 

### Method

#### Participants

Eighty-three further children of the same primary school of Experiment 1 participated: 21 children (of which 11 females) of grade 2 (mean age: 7 years, 7 months), 28 (18 females) of grade 3 (mean age: 8 years, 7 months), and 34 (19 females) of grade 4 (mean age: 9 years, 7 months). We obtained written informed consent from all children’s parents. The children were all native speakers of Dutch and were instructed according to the same curricula as the children of Experiment 1. The children of grade 2 had received 37 weeks of formal reading and writing instruction in grade 1 and 9 weeks in grade 2. Children of grade 1 could not be included in this experiment because they did not know enough words that could be used to form a variety of pseudohomophones.

In each grade, a group of more and less advanced spellers was formed based on a median split on the spelling scores of the first month courses (testing was done beginning of November, so we used the scores on the school report of October). This way, children of each grade were divided into a group of children with below and above average orthographic knowledge. 

In order to demonstrate the comparability of the children across grades, we compared the scores on a normed and standardized test for spelling and mathematics (in Dutch: *Leerlingvolgsysteem*). This test measures a child’s learning progress and is administered to each grade at the beginning, middle and end of the school year. This way, it is tested whether each child in each grade reached the attainment goals that are specified for that specific grade. These attainment goals have to be reached by all schools in Belgium. We compared the mean scores on spelling and mathematics on the last test. The results showed no significant differences between grades as shown by an independent samples t-test (*p* > .31). Similarly, there were no significant differences in spelling scores across grades (*p* > .53). These results showed that the children in each grade reached the attainment goals and therefore, the comparability of the children’s scholastic abilities across grades.

#### Stimuli

The 12 pseudohomophone-control pairs of Experiment 1 were used again, together with 72 fillers words (similar length, *M* = 4.36 letters, *SD* = 0.81). Contrary to Experiment 1, we now presented all pseudohomophones and controls to each subject. We presented the stimulus list in two separate blocks of 48 stimuli, to avoid concentration issues. Each block contained 6 pseudohomophones and 6 control misspellings of different base words and 36 fillers. 

#### Procedure

The proofreading test was administered simultaneously to all the children of a class. They could not see each other’s forms and children who were sitting next to each other received different lists. Instructions were the same as in Experiment 1. Between proofreading the two lists, there was a short break of about an hour. 

To check the orthographic knowledge of the children, a spelling test of the 12 base words was administered approximately 3 to 4 hours after completion of the second proofreading block. Each base word was spoken out loud and children wrote down every word. 

### Results

#### Spelling results

**Figure 2:**
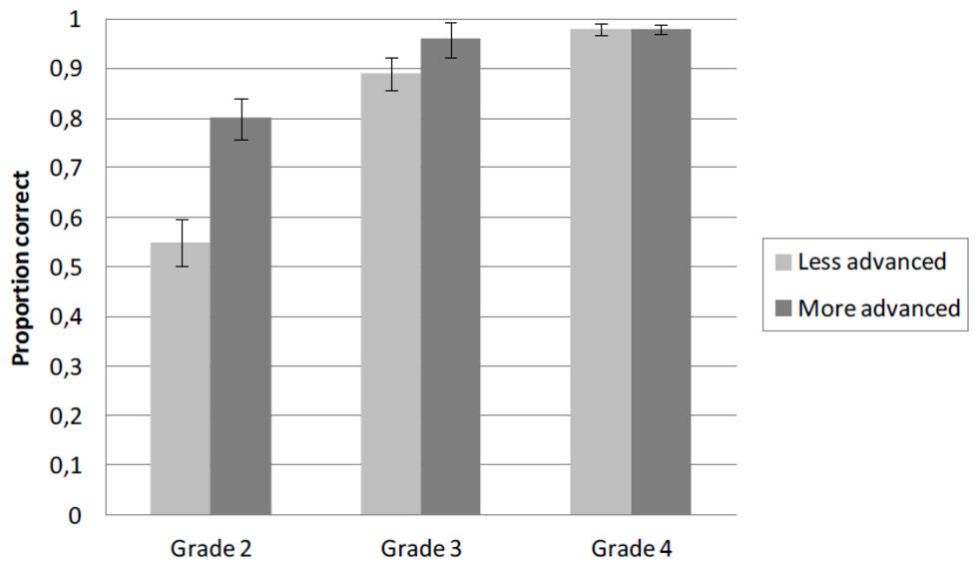
Mean proportion correctly spelled base words for less and more advanced spellers as a function of Grade in Experiment 2. Error bars show standard errors. Proportions are based on 12 items per condition.

We analyzed spelling accuracy in ANOVAs with participants (F_1_) and items (F_2_) as the random variables. The results showed that most children could write the words down correctly. Only words that were spelled entirely correctly (not just the target grapheme) were calculated as a correct response. No words had to be excluded because of non-readable writing. Mean accuracy was 0.68 in the second grade, 0.93 in the third grade, and 0.98 in the fourth grade. An ANOVA with Grade (three levels: grade 2, 3, and 4) and Orthographic knowledge (two levels: more and less advanced) as the independent variables indicated that accuracy scores improved between grades [*F*_1_(2,77) = 52.52, *p* < .001; *F*_2_(2,22) = 19.83, *p* < .001]. Also, more advanced spellers had higher scores than less advanced spellers [*F*_1_(1,77) = 19.12, *p* < .001; *F*_2_(1,11) = 15.61, *p* < .001]. Most importantly, the interaction between these two factors was significant [*F*_1_(2,77) = 9.01, *p* < .001; *F*_2_(2,22) = 9.99, *p* < .001]. This interaction originated from the fact that more advanced spellers (*M* = 0.80) scored significantly better than less advanced spellers (*M* = 0.55) in the second grade [*F*_1_(1,77) = 28.52, *p* < .001; *F*_2_(1,11) = 13.69, *p* < .001]. In grade 3, this difference between more (*M* = 0.96) and less advanced spellers (*M* = 0.89) was much smaller and only significant in the analysis by items [*F*_1_(1,77) = 2.55, *p* = .11,; *F*_2_(1,11) = 5.86, *p* < .05]. No differences between groups (both *M* = .98) were observed in grade 4. A graph of the interaction and mean scores for more and less advanced spellers is presented in Figure 2. 

#### Proofreading results

**Figure 3:**
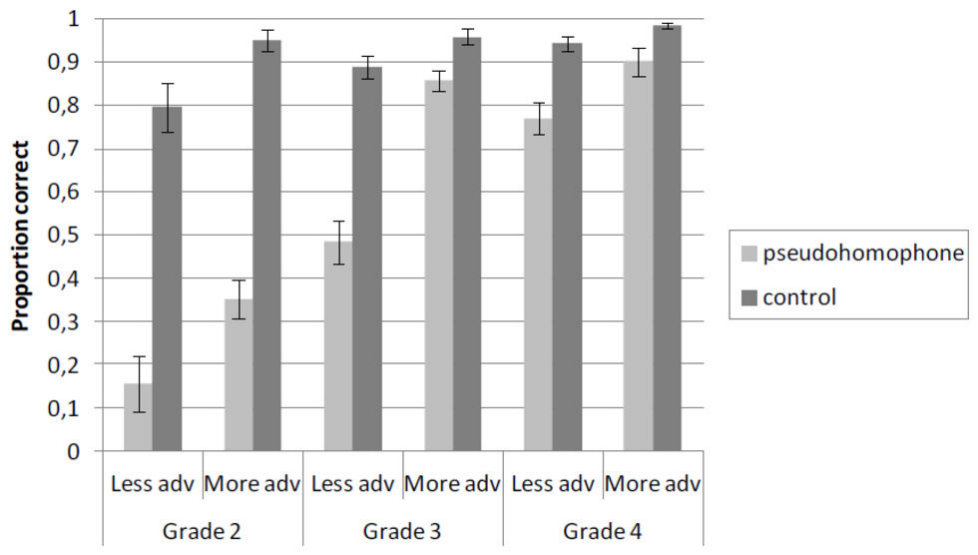
Mean proportion correctly classified pseudohomophone and control misspellings as a function of Grade in Experiment 2. Error bars show standard errors. Proportions are based on 12 items per condition.

An ANOVA was run with Grade (three levels: grade 2, 3, and 4), Word type (two levels: pseudohomophone vs. control), and Orthographic knowledge (two levels: more and less advanced) as the independent variables. For each child and base word, we verified whether the correct spelling was written down in the spelling test. If this was not the case, the pseudohomophone and its corresponding control misspelling were removed from the data. Following this procedure, 32% of the trials were removed in grade 2, 7% in grade 3, and 2% in grade 4. Subject and item means of the proportion correctly identified misspellings were calculated. Mean accuracy by Word type, Grade and Orthographic knowledge is depicted in Figure 3. 

The analysis yielded significant main effects of Word type [*F*_1_(1,77) = 171.79, *p* < .001; *F*_2_(1,20) = 93.52, *p* < .001], Grade [*F*_1_(2,77) = 33.60, *p* < .001; *F*_2_(2,40) = 80.36, *p* < .001], and Orthographic knowledge [*F*_1_(1,77) = 21.94, *p* < .001; *F*_2_(1,20) = 80.53, *p* < .001]. Also, there were significant interactions between Word type and Grade [*F*_1_(2,77) = 29.50, *p* < .001; *F*_2_(2,40) = 44.07, *p* < .001], and between Word type and Orthographic knowledge [*F*_1_(1,77) = 8.33, *p* < .01; *F*_2_(1,20) = 18.33, *p* < .001]. Most importantly, there was a significant three-way interaction of Word type, Grade, and Orthographic knowledge [*F*_1_(2,77) = 2.94, *p* = .06; *F*_2_(2,40) = 8.18, *p* < .01]. This interaction is depicted in Figure 3 and showed that the pseudohomophone effect decreased much more between grades 2 and 3 for more advanced spellers, than for less advanced spellers [*F*_1_(1,77) = 4.83, *p* < .05; *F*_2_(1,20) = 4.21, *p* = .05]. More advanced spellers already performed at the same level in grade 3 as in grade 4 [*F*s < 1], whereas less advanced spellers showed a decreasing pseudohomophone effect between grades 3 and 4 [*F*_1_(1,77) = 9.25, *p* < .01; *F*_2_(1,20) = 22.85, p < .001]. 

Planned comparisons showed a strong pseudohomophone effect in grade 2 for more advanced [*F*_1_(1,77) = 75.17, *p* < .001; *F*_2_(1,20) = 122.07, *p* < .001], and for less advanced spellers [*F*_1_(1,77) = 75.48, *p* < .001; *F*_2_(1,20) = 55.07, *p* < .001]. Strong pseudohomophone effects were also observed for less advanced spellers in grade 3 [*F*_1_(1,77) = 48.51, *p* < .001; *F*_2_(1,20) = 47.17, *p* < .001]. The effect for more advanced spellers in grade 3 was only significant in the analysis by items [*F*_1_(1,77) = 2.95, *p* = .09; *F*_2_(1,20) = 8.18, *p* < .01]. Indeed, the pseudohomophone effect was stronger for less advanced than for more advanced spellers in grade 3 [*F*_1_(1,77) = 13.77, *p* < .001; *F*_2_(1,20) = 25.92, *p* < .001]. In grade 4, the pseudohomophone effect was significant for less advanced spellers [*F*_1_(1,77) = 9.92, *p* < .01; *F*_2_(1,20) = 15.59, *p* < .001], and for more advanced spellers in the item analysis [*F*_1_(1,77) = 2.21, *p* = .14; *F*_2_(1,20) = 4.21, *p* = .05]. The test for a stronger pseudohomophone effect for less advanced than for more advanced spellers in grade 4 was only significant in the analysis by items [*F*_1_(1,77) = 1.38, *p* = .24; *F*_2_(1,20) = 9.00, *p* < .01].

In sum, the results of Experiment 2 show that, as in Experiment 1, fewer pseudohomophones were detected than control misspellings, providing further support for the important role of phonological recoding in proofreading. Pseudohomophone effects were most pronounced in grade 2, and then gradually decreased, but not completely disappeared, in grades 3 and 4. Moreover, this pseudohomophone effect was modulated by the degree of orthographic knowledge of children. Although there was no difference in the size of the pseudohomophone effect between more and less advanced spellers in grade 2, this pseudohomophone effect was much stronger for less advanced than for more advanced spellers in grade 3. From grade 3 on, more advanced spellers already reached the same level as in grade 4. Less advanced spellers reached this level only later. 

## General Discussion

This cross-sectional study examined the role of phonological recoding in the early stages of reading development, using the pseudohomophone effect as a marker of automatic phonological recoding. In contrast with previous studies, analyses only included words for which an orthographic choice task or spelling task ensured that children actually knew the spelling. As such, any pseudohomophone effect may be unambiguously related to automatic phonological recoding. Furthermore, this allowed us to investigate whether children also use their orthographic knowledge optimally in a reading task such as proofreading. In Experiment 1, children in grades 1, 2, and 3 detected more control misspellings than pseudohomophone misspellings. This pseudohomophone effect was more pronounced in grade 1 than it was in grade 2, but there was no difference between grades 2 and 3. In Experiment 2, this decrease in phonological recoding effects as a function of grade was replicated with children in grades 2, 3, and 4. Additionally, spelling expertise modulated the strength of the pseudohomophone effect across grades. There was no difference in the size of the pseudohomophone effect between more and less advanced spellers in grade 2, while in grade 3, the pseudohomophone effect was much stronger for less advanced than for more advanced spellers. From grade 3 on, more advanced spellers already reached the same level as in grade 4, but less advanced spellers reached this level only later. 

It should be noted that the results of Experiments 1 and 2 differ with respect to the evolution of pseudohomophone effects. In Experiment 1, performance on pseudohomophones did not increase from grades 2 to 3, whereas it did in Experiment 2. This difference is likely to be due to the fact that the participants of Experiment 1 were already in the second term of the school year when being tested, while the subjects of Experiment 2 were in the first term. Thus, the children of the first experiment had already received more reading and spelling courses relative to the participants of Experiment 2 in the same grade. Still, the pseudohomophone effect for children in Experiment 2 diminished from grade 3 to grade 4, but this was only the case for less advanced spellers. 

Also note that performance on pseudohomophones increased gradually with grade (e.g., Experiment 1: from .29 in grade 1 to .81 in grade 3), whereas performance on control misspellings was high from grade 1 on (.84). These (near) ceiling effects for controls follow naturally from the way children (and adults) process words and are present in most other studies with similar research questions and similar designs (e.g., 19,21,23,26). A control misspelling (e.g., *geim* for the base word *geit* ‘goat’) has no representation in lexical memory, nor does it have the same pronunciation as an existing word, so that it is relatively easy to correctly mark this item as a misspelling, and this already from grade 1 on. The good performance on control misspellings shows that children knew the orthographic word forms, and this was also confirmed in the orthographic choice and spelling tests. The performance on pseudohomophones was lower than this overall spelling level indicating that children phonologically recoded the words (i.e., the phonology of a pseudohomophone can activate the orthographic representation of its base word, leading to an acceptance of the pseudohomophone as a correct word). The strict matching procedure for pseudohomophones and control misspellings ensured that phonological overlap with a word was the critical difference between pseudohomophones and controls. 

The present pattern of results in Dutch showing decreasing phonological recoding effects during proofreading as a function of increasing reading proficiency is in agreement with the results from previous studies on other languages (e.g., English: [16, 26]; French: [18, 23]). For instance, Sprenger-Charolles et al. [18] observed strong pseudohomophone effects for beginning French readers in a semantic categorization task and these effects diminished from grade 3 onwards. Interestingly, the present pseudohomophone effect already decreased from grade 1 onwards. This may indicate that Dutch readers develop and use orthographic knowledge earlier in reading development than French readers and is likely to be related to cross-language differences in orthographic consistency. This is in accordance with the developmental model of Share [9] which states that the phonological procedure provides the basic mechanism for acquiring word-specific orthographic representations. However, note that task differences in Sprenger-Charolles et al. and the present study and the fact that Sprenger-Charolles et al. did not remove unknown words from the analyses may also contribute to the difference in results. Still, Grainger et al. [23] also tested pseudohomophones and their pseudohomophone effects in beginning readers are generally smaller than the ones found in Dutch. Although there are also task differences present here, this may again indicate a somewhat stronger influence of phonological recoding in early stages of Dutch reading. 

Our cross-sectional results for Dutch supplement earlier results in Dutch language research on the pseudohomophone effect by Bosman and de Groot [21] and the word length effect by Martens and de Jong [34]. Not only did we replicate Bosman and de Groot’s pseudohomophone effect for first graders, we also observed a diminishing pseudohomophone effect with increasing proficiency in Dutch. The pseudohomophone effect decreased more slowly for children with less orthographic knowledge than for children with more orthographic knowledge. In Bosman and de Groot’s study, less advanced readers showed stronger pseudohomophone effects than more advanced readers at the end of grade 1, whereas we only observed such a difference at the beginning of grade 3. There may be several reasons for this difference. First, there may be a difference between groupings based on reading versus spelling knowledge. However, Bosman and de Groot reported a strong, significant correlation (*r* = .50) between reading and spelling proficiency (see also 41). Second, the strong pseudohomophone effect reported for more advanced readers in Bosman and de Groot might not have been that strong if the data were analyzed differently. They used an orthographic choice task in which children have a 50% chance of choosing the correct response if they did not know the right answer. In addition, items for which children did not know the spelling were not filtered out from the analyses. As the results of the orthographic choice task were weaker for less advanced (*M* = .86) than for more advanced readers (*M* = .96), it might be that analyzing the results in a different way tuned down the strong pseudohomophone effect difference between more and less advanced readers. 

The results are in accordance with reading acquisition accounts that assume an important role for phonological recoding in beginning reading (e.g., 9-11). It seems that in the beginning of reading development, phonological recoding strongly affects children’s word reading, because even though a spelling test had shown that children had orthographic knowledge of the words, they still failed to reject pseudohomophone misspellings. This suggests that they may not use their orthographic knowledge optimally in a reading task such as proofreading and indicates the strong involvement of phonological recoding. They face two conflicting responses: based on phonological recoding, the pseudohomophone should be a word, whereas it should be a nonword based on their orthographic knowledge. More experienced spellers and readers grow less dependent on phonological recoding in their decision to mark misspellings. They increasingly master consolidated word-specific orthographic knowledge and as a result, the more experienced spellers and readers can correctly reject pseudohomophones. 

Bosman and de Groot [21] put forward spelling verification as the basic mechanism for detecting misspellings. As discussed earlier, the verification hypothesis assumes that in order to identify a pseudohomophone as a misspelling, readers compare their knowledge of the correct spelling with the spelling of the stimulus. In Bosman and de Groot’s study, effective spelling verification was positively related to reading skill, but the differential performance of more advanced readers on for example proofreading lists versus stories showed that spelling verification is not yet stable in beginning readers. Bosman and de Groot assumed that the activation of phonology is a primary constraint in all tasks, but that task context can add additional constraints that work against successful verification. They also concluded that, based on their results with beginning readers and Van Orden and colleagues’ results [22,42] it may be legitimate to infer that phonological processing underlies the reading of beginning and skilled readers alike. Phonological recoding indeed has a long lasting influence on reading (e.g., 18) and there is ample evidence that phonological information is automatically activated in adult reading, both in reading in a native language (e.g., [22,43–45]; for a review of evidence supporting a strong phonological theory, see 46) and in reading in a second language [47]. For instance, in Sprenger-Charolles et al. [18], significant pseudohomophone effects were still observed in grade 4. This last result is similar to the present results, for we observed that the children in grades 3 and 4 still showed a small pseudohomophone effect. This means that phonological recoding still has an influence in proofreading, even for these familiar words for which they acquired sufficient orthographic knowledge as revealed by the spelling test. It seems that phonological recoding and the use of orthographic whole-word knowledge are interactive processes during reading. 

To conclude, the present results provide new insights into the development of phonological recoding in Dutch readers. As such, it adds to the language variety in the reading acquisition literature. Phonological recoding was found to have a significant influence in error detection in first and second grade children, even for words for which it was shown that children know the spelling. The effect of phonological recoding diminished, but remained significant, as readers become more advanced. 

## Supporting Information

Appendix S1 Table S1 - List of the base words, pseudohomophones and control misspellings that were used in Experiments 1 and 2. Table S2 - List of the filler words used in Experiments 1 and 2.
(DOCX)
