# Supplementary material for: Phonological Recoding in Error Detection: A Cross-sectional Study in Beginning Readers of Dutch
Source: PLoS One. 2013 Dec 30;8(12):e85111. doi: 10.1371/journal.pone.0085111 (PMC3875550; doi:10.1371/journal.pone.0085111)
Supplement: Appendix S1 — Table S1 - List of the base words, pseudohomophones and control misspellings that were used in Experiments 1 and 2. Table S2 - List of the filler words used in Experiments 1 and 2. (DOCX) [file pone.0085111.s001.docx]

Appendix A

In this Appendix, we provide some more details on the studies we discussed in our email.

Bosman and De Groot (1996) found that in a **proofreading task** in which unconnected lists of words were presented (Experiment 1), Dutch children detected fewer pseudohomophone misspellings (i.e., nonwords that sound like real words) than control misspellings (i.e., nonwords that do not sound like real words). As in our study, the children proofread lists of unconnected words, making the experimental conditions highly similar to ours. The table below illustrates that proportions correct to controls were highly similar to the numbers in our study. Furthermore, first graders in Bosman and De Groot (1996) also performed a lexical decision task (Experiment 3) and semantic categorization task (Experiment 4) in which pseudohomophone and control misspellings were presented. Again, performance on controls was at ceiling.

|  |  |  | Pseudo-homophone | Control nonword |
| --- | --- | --- | --- | --- |
| Proofreading | Grade 1 | Less adv | .16 | .82 |
|  |  | More adv | .67 | .84 |
| Lexical decision | Grade 1 | Less adv | .39 | .93 |
|  |  | More adv | .70 | .89 |
| Semantic categorization | Grade 1 | Less adv | .32 | .98 |
|  |  | More adv |  |  |

V. Coltheart, Laxon, Rickard, and Elton (1988) investigated phonological recoding in reading. In Experiment 2, English-speaking children aged 8, 9 or 11 years **judged whether printed sentences were correct or no**t. They presented incorrect sentences that included a pseudohomophone (e.g. *wair* [wear]; *The girl will wair a dress*) or an orthographic control nonword (e.g., *waur*; *She will waur a hat*). Significant pseudohomophone effects were observed. As can be seen in the table below, proportion correct responses for controls were very similar to the ones in our study.

|  |  | Pseudo-homophone | Control nonword |
| --- | --- | --- | --- |
| Competent readers | 11 years | .84 | .97 |
|  | 9 years | .89 | .98 |
|  | 8 years | .81 | .92 |
| Poor readers | 11 years | .64 | .82 |
|  | 9 years | .64 | .85 |

In Grainger, Lété, Bertand, Dufau, and Ziegler (2012), French children in grades 1 to 5 performed a **lexical decision task** in which they classified letter strings as words or nonwords. Nonwords included pseudohomophones (e.g., *trane* [train]) and orthographic controls (e.g., *tarpe*). Overall, it was harder to classify pseudohomophones as nonwords (proportion correct responses .56) than to classify controls as nonwords (.82). Mean proportion of correct answers per grade is again very high and similar to the numbers in our study.

|  | Pseudo-homophone | Control nonword |
| --- | --- | --- |
| Grade 1 | .22 | .67 |
| Grade 2 | .41 | .74 |
| Grade 3 | .64 | .84 |
| Grade 4 | .73 | .94 |
| Grade 5 | .77 | .93 |

Goswami, Ziegler, Dalton, and Schneider (2001) used a **lexical decision task** (Experiment 2) to compare the development of phonological recoding in 8- and 9-year-old children learning to read English and German. Reading of pseudhomophone nonwords (e.g., English *faik* [fake]) was compared to the reading of control nonwords that were orthographically and phonologically similar to real words but that were not phonologically identical to real words (e.g., *dake*). As in the previous studies mentioned above, and similar to our results, children performed at ceiling level for the controls.

| Proportion correct | Pseudo-homophone | Control nonword |
| --- | --- | --- |
| English | .80 | .83 |
| German | .60 | .91 |

In Johnston, Thompson, Fletcher-Flinn, and Holligan (1995), English-speaking 8- and 11-year-olds performed a **proofreading/lexical decision task** (Experiment 1) in which pseudohomophones (e.g., *poast* [post]) and control nonwords (*loast*) were included. Only 8-year-olds who received a phonics instruction showed a pseudohomophone effect. More importantly, performance for the controls was very high.

| Proportion correct |  | Pseudo-homophone | Control nonword |
| --- | --- | --- | --- |
| 8-year-olds | Phonics | .80 | .91 |
|  | Nonphonics | .88 | .91 |
| 11-year-olds | Phonics | .94 | .95 |
|  | Nonphonics | .95 | .96 |
